# Risk factors for disease progression in Japanese patients with COVID-19 with no or mild symptoms on admission

**DOI:** 10.1186/s12879-021-06574-x

**Published:** 2021-08-21

**Authors:** Toshifumi Ninomiya, Kohei Otsubo, Teppei Hoshino, Mototsugu Shimokawa, Megumi Nakazawa, Yoriko Sato, Hironori Mikumo, Satoru Kawakami, Shun Mizusaki, Yusuke Mori, Hidenobu Arimura, Yuko Tsuchiya-Kawano, Koji Inoue, Yujiro Uchida, Yoichi Nakanishi

**Affiliations:** 1 Department of Respiratory Medicine, Kitakyushu Municipal Medical Center, 2-1-1 Bashaku, Kokurakita-ku, Kitakyushu, Fukuoka 802-0077 Japan; 2 Department of General Internal Medicine, Kitakyushu Municipal Medical Center, 2-1-1 Bashaku, Kokurakita-ku, Kitakyushu, Fukuoka 802-0077 Japan; 3 Department of Internal Medicine, Kitakyushu City Yahata Hospital, 2-6-2, Ogura, Yahatahigashi-ku, Kitakyushu, Fukuoka 805-8534 Japan; 4 Department of Biostatistics, Yamaguchi University Graduate School of Medicine, 1-1-1 Minamikogushi, Ube-shi, Yamaguchi 755-8505 Japan; 5 Department of Respiratory Medicine, JCHO Kyushu Hospital, 1-8-1 Kishinoura, Yahatanishi-ku, Kitakyushu, Fukuoka 806-8501 Japan; 6 Kitakyushu City Hospital Organization, 1-35 Furusenbamachi, Kokurakita-ku, Kitakyushu, 802-0082 Japan

**Keywords:** COVID-19, Risk factors, Disease progression

## Abstract

**Background:**

Although the risk factors for coronavirus disease 2019 (COVID-19) mortality have been identified, there is limited information about the risk factors for disease progression after hospitalization among Japanese patients with COVID-19 exhibiting no or mild symptoms.

**Methods:**

All 302 consecutive patients who were admitted to our institutions and diagnosed with COVID-19 between March and December 2020 were retrospectively assessed. Ultimately, 210 adult patients exhibiting no or mild symptoms on admission were included in the analysis. They were categorized into the stable (no oxygen needed) and worsened (oxygen needed) groups, and their characteristics and laboratory data were compared.

**Results:**

Among 210 patients, 49 progressed to a severe disease stage, whereas 161 did not. The mean patient age was 52.14 years, and 126 (60.0%) patients were male. The mean body mass index (BMI) was 23.0 kg/m^2^, and 71 patients were overweight (BMI ≥ 25 kg/m^2^). Multivariate logistic analysis showed that old age, overweight, diabetes mellitus (DM), and high serum ferritin levels were independent risk factors for disease progression.

**Conclusions:**

Clinicians should closely observe patients with COVID-19, especially those with risk factors such as old age, overweight, DM, and high serum ferritin levels, regardless of whether they have no or mild symptoms.

## Background

In December 2019, coronavirus disease 2019 (COVID-19), which is caused by severe acute respiratory syndrome coronavirus 2 (SARS-CoV-2), emerged in Wuhan, China, and subsequently spread globally, leading to a pandemic. In the early stage of the COVID-19 pandemic in Japan, most patients were hospitalized with or without symptoms. However, medical institutions were overwhelmed by the transmission of infection within a few months, and patients were recommended hospitalization, admission to a dedicated hotel, or home care on the basis of their symptoms and hospital availability at the discretion of the local health center and physicians. Although approximately 80% of COVID-19 cases are mild or asymptomatic, 20% develop pneumonia, and some progress to respiratory failure, acute respiratory distress syndrome, and multiple organ failure [1–4]. The reported risk factors for poor prognosis in COVID-19 include old age, male sex, obesity, comorbidities such as cardiovascular disease and diabetes mellitus (DM), and biomarkers, including C-reactive protein (CRP) level, lactate dehydrogenase (LDH) level, interleukin (IL)-6 level, ferritin level, D-dimer level, and neutrophil-to-lymphocyte ratio (NLR) [5–10]. However, little is known about the risk factors for developing severe disease after hospitalization among Japanese patients with COVID-19 exhibiting no or mild symptoms. Since March 2020, our two institutions of the Kitakyushu City Hospital Organization have accepted the largest number of patients with COVID-19 in Kitakyushu City. At our institution, patients without any symptoms or those with mild symptoms were only monitored or administered symptomatic treatment, such as antipyretics and antitussives, whereas oxygen supplementation was administered to patients who presented with low oxygen saturation on pulse oximetry (SpO_2_ ≤ 93% at rest). We have encountered some patients who developed respiratory failure and required oxygen administration or mechanical ventilation after hospitalization despite exhibiting no or mild symptoms on admission. Thus, this study aimed to retrospectively evaluate the risk factors for disease progression in patients with COVID‐19 presenting with no or mild symptoms on admission.

## Methods

### Study design and participants

This multicenter, retrospective study was performed at Kitakyushu Municipal Medical Center and Kitakyushu City Yahata Hospital in Fukuoka, Japan. All 302 consecutive patients who were admitted to our institutions and diagnosed with COVID-19 between March 23, 2020, and December 31, 2020, were retrospectively assessed. Patients diagnosed with COVID-19 using polymerase chain reaction or antigen testing of nasopharyngeal swabs or sputum for SARS-CoV-2 and either did not exhibit any symptoms or had mild symptoms on admission were enrolled in this study. Pediatric patients (< 15 years old) and patients for whom data on specified physical characteristics (height, weight, and comorbidities) and laboratory tests (complete blood count, CRP, LDH, ferritin, and D-dimer) were unavailable were excluded. The follow-up period ended on the day when patients recovered and were discharged, were transferred to an advanced medical institution to receive intensive care, or died.

### Clinical analysis

The following details were collected from the patient’s medical records: age (per 10 years), sex, body mass index (BMI), comorbidities (asthma, hypertension, cardiovascular disease, DM, and interstitial lung disease), respiratory status, laboratory data (white blood cell count, neutrophil count, lymphocyte count, hemoglobin, CRP level, LDH level, ferritin level, and D-dimer level at admission), history of remdesivir and systemic corticosteroid usage before oxygen supplementation, and outcomes. All methods were performed in accordance with the relevant guidelines and regulations.

### Definition of disease status

We classified the patients into the following two groups according to their respiratory status during the disease course: (1) worsened group, which comprised patients who needed oxygen administration because of pneumonia and low oxygen saturation on pulse oximetry (SpO_2_ ≤ 93% at rest) and (2) stable group, which comprised patients with no or mild symptoms who did not need oxygen administration.

### Statistical analysis

The clinical characteristics of the patients were summarized using descriptive statistics and were compared using Fisher’s exact test for categorical data and Student’s *t*-test for continuous data. The risk factors for disease progression were evaluated using a logistic regression model. Multivariate logistic regression was performed using the backward elimination method. A P-value of < 0.05 was considered statistically significant. Complete case analysis was adopted for all statistical analysis, and JMP Pro version 15 software for conducting statistical analysis (SAS Institute, Cary, NC, USA).

## Results

### Patient characteristics and clinical course

**Fig. 1:**
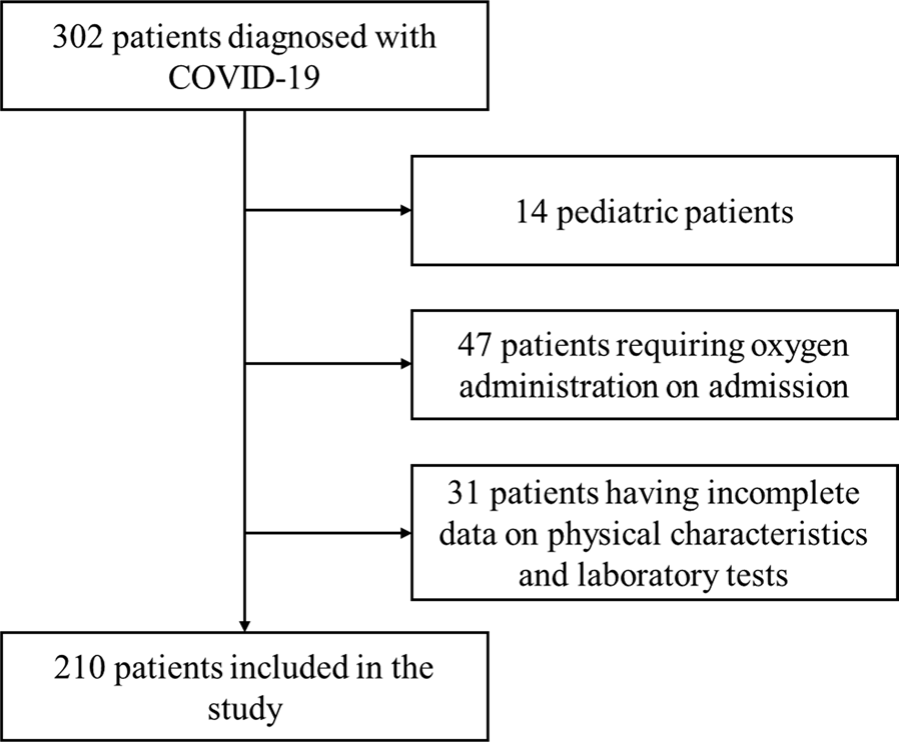
Flowchart of patient enrollment. COVID‐19, coronavirus disease 2019

We excluded 14 pediatric patients, 47 patients who already required oxygen supplementation on admission, and 31 patients with incomplete data on specified physical characteristics and laboratory tests. Ultimately, 210 of the 302 patients were included in the analysis (Fig. 1). During hospitalization, 49 patients required oxygen supplementation (worsened group), whereas 161 patients remained stable until discharge (stable group). Among the 49 patients who exhibited disease progression, 4 died, but the remaining 45 recovered. The clinical characteristics of the patients on admission are shown in Table 1. The mean age of the patients was 52.14 years (15–98 years), and 126 (60.0%) of them were male. The mean BMI was 23.0 kg/m^2^, and 71 patients were overweight (BMI ≥ 25.0 kg/m^2^). The major comorbidities were hypertension (24.8%), asthma (12.4%), DM (11.0%), and cardiovascular disease (11.0%). None of the patients were administered remdesivir or systemic corticosteroids prior to oxygen supplementation.

**Table 1:** Clinical characteristics of patients with COVID-19 on admission

Characteristics	Total (n = 210)	Worsened (n = 49)	Stable (n = 161)	P value
Age, years	52.14 ± 20.14	63.47 ± 13.39	48.69 ± 20.58	< 0.001
Sex, n (%)				0.013
Male	126 (60.0)	37 (75.5)	89 (55.3)	
Female	84 (40.0)	12 (24.5)	72 (44.7)	
BMI, n (%)				< 0.001
< 25 kg/m^2^	139 (66.2)	20 (40.8)	119 (73.9)	
≥ 25 kg/m^2^	71 (33.8)	29 (59.2)	42 (26.1)	
Comorbidities, n (%)				
Hypertension	52 (24.8)	25 (51.0)	27 (16.8)	< 0.001
Asthma	26 (12.4)	6 (12.2)	20 (12.4)	> 0.99
Diabetes mellitus	23 (11.0)	11 (22.5)	12 (7.5)	0.0071
Cardiovascular disease	23 (11.0)	10 (20.4)	13 (8.1)	0.033
Interstitial lung disease	5 (2.4)	0 (0.0)	5 (3.1)	0.59
Laboratory data				
WBC, 10^9^/L	5.19 ± 2.07	5.33 ± 2.36	5.15 ± 1.94	0.58
NLR	4.21 ± 8.26	4.45 ± 4.18	4.32 ± 5.35	0.21
Hemoglobin, g/dL	14.07 ± 6.22	13.88 ± 1.93	14.13 ± 1.70	0.37
CRP, mg/dL	1.78 ± 7.41	3.55 ± 3.89	1.24 ± 2.30	< 0.001
LDH, U/L	206.19 ± 68.81	245.35 ± 3.89	194.27 ± 62.54	< 0.001
Ferritin, ng/mL	282.74 ± 303.02	507.24 ± 437.22	214.41 ± 204.81	< 0.001
D-dimer, µg/mL	1.31 ± 7.87	1.43 ± 2.98	1.28 ± 4.14	0.81

Categorical data are presented as numbers (percentages) and were analyzed using Fisher’s exact test. Continuous data are presented as mean ± standard deviation and were analyzed using Student’s *t*-test. P < 0.05 was considered statistically significant

*BMI* body mass index, *WBC* white blood cell, *NLR* neutrophil-to-lymphocyte ratio, *CRP* C-reactive protein, *LDH* lactate dehydrogenase

### Risk factors for disease progression

Table 2 summarizes the univariate and multivariate analyses results of the risk factors for disease progression. In univariate analysis, old age, male sex, BMI ≥ 25 kg/m^2^, hypertension, cardiovascular disease, DM, CRP level, LDH level, creatinine level, and ferritin level were significantly associated with disease progression. In multivariate analysis, old age (odds ratio [OR] 1.538; 95% confidence interval [CI] 1.219–1.949; P < 0.001), overweight (OR 3.260; 95% CI 1.463–7.265; P = 0.0038), DM (OR 2.851; 95% CI 1.056–7.698; P = 0.039), and high serum ferritin levels (OR 1.002; 95% CI 1.001–1.004; P < 0.001) were found to be independent risk factors for disease progression.

**Table 2:** Risk factors for disease progression in patients with COVID-19

Factor	Univariate		Multivariate	
	OR (95% CI)	P value	OR (95% CI)	P value
Age (per 10 years)	1.495 (1.243–1.808)	< 0.001	1.538 (1.219–1.949)	< 0.001
Sex	2.494 (1.212–5.132)	0.013		
Overweight (BMI ≥ 25)	4.108 (2.103–8.025)	< 0.001	3.260 (1.463–7.265)	0.0038
Asthma	0.984 (0.371–2.606)	0.97		
Hypertension	5.170 (2.577–10.37)	< 0.001		
Cardiovascular disease	2.919 (1.191–7.156)	0.019		
Diabetes	3.594 (1.473–8.773)	0.0050	2.851 (1.056–7.698)	0.039
NLR	1.057 (0.970–1.153)	0.21		
CRP	1.272 (1.132–1.430)	< 0.001		
LDH	1.010 (1.005–1.015)	< 0.001		
Creatinine	9.551 (2.327–39.20)	0.0017		
Ferritin	1.003 (1.002–1.004)	< 0.001	1.002 (1.001–1.004)	< 0.001
D-dimer	1.009 (0.936–1.088)	0.81		

Risk factors associated with disease severity were analyzed using a logistic regression model. P < 0.05 was considered statistically significant

*OR* odds ratio, *CI* confidence interval, *BMI* body mass index, *NLR* neutrophil-to-lymphocyte ratio, *CRP* C-reactive protein, *LDH* lactate dehydrogenase

## Discussion

The risk factors for COVID-19 mortality have been extensively reported worldwide. Although reports on such risk factors in Japanese patients are limited, a nationwide COVID-19 inpatient registry, COVID-19 Registry Japan (COVIREGI-JP), was created in March 2020. Matsunaga et al. identified the clinical epidemiological characteristics of hospitalized patients with COVID-19 registered in COVIREGI-JP and reported that the mortality rate of these patients was 7.5%, which was lower than that in other countries (~ 15–25%) [4, 11, 12]. However, some Japanese patients with COVID-19 developed respiratory failure and required oxygen administration or mechanical ventilation despite having no or mild symptoms on admission. Hence, this study investigated the clinical characteristics of patients with COVID-19 with no or mild symptoms on admission. The results revealed that old age, overweight, DM, and high serum ferritin level were risk factors for developing a severe form of COVID-19 after hospitalization. Although these risk factors are mentioned in a previous COVIREGI-JP cohort study, our study was novel in that it was limited to patients without any symptoms or those having mild symptoms on admission and demonstrated that these are statistically significant risk factors for disease progression. Asymptomatic patients with COVID-19 or those with mild symptoms are often monitored at home or in a hotel rather than being hospitalized. Therefore, the clinical implication of our study is that whether these patients have the risk factors for disease progression identified in this study will help determine their monitoring facility (hospital, hotel, or home) at the time of diagnosis. Thereby, physicians can carefully follow-up on patients with risk factors for disease progression and provide immediate treatment on worsening of symptoms.

The OR for disease progression in patients who were overweight was 3.260, which was the highest among statistically significant risk factors in this study. Obesity affects the respiratory function through different mechanisms, such as respiratory compromise, chronic inflammation, and complications. Obesity is also associated with pulmonary restriction, ventilation–perfusion mismatch, respiratory muscle fatigue, and sleep apnea syndrome; thus, patients with obesity have a background of hypoxemia and are more prone to respiratory failure [13]. These patients also have higher expression levels of tumor necrosis factor α, IL-1, and IL-6. These proinflammatory cytokines increase the inflammatory response and abnormal T cell response, causing drastic lung injury and severe pneumonia [14]. When chronic hypoxia stimulates the hypoxia-inducible factor, detrimental effects occur on the immune system, as well as cytokine storm, which is associated with the poor outcomes of COVID-19 [15]. Overweight and obesity are risk factors of severe illness in patients with COVID-19 [16–18]. Therefore, one factor that can explain the low COVID-19 mortality rate in Japan is that this country has a small obese population compared with Western countries. Our study also revealed that DM was a statistically significant risk factor for disease progression. Hyperglycemia causes immune dysfunction, including impaired neutrophil function, antioxidant system function, and humoral immunity; thus, patients with DM with COVID-19 have a high risk of disease progression [19, 20]. Furthermore, high serum ferritin levels at admission were significantly associated with the disease progression of COVID-19. Because serum ferritin is an iron-storage protein that regulates cellular oxygen metabolism, it is used as a marker for iron overload disorders, including hemochromatosis and hemosiderosis, which were not identified in patients enrolled in this study. It is also an immunomodulatory molecule with both immunosuppressive and proinflammatory functions that cause cytokine storms [21]. Elevated ferritin serum levels reportedly correlate with disease severity in patients with COVID-19 [22–24]. Therefore, patients with elevated serum ferritin levels are more likely to develop cytokine storms and subsequently, severe respiratory failure.

Recent studies have shown that high levels of D-dimer and NLR are risk factors for mortality in patients with COVID-19 [5, 10]. However, these factors were not significantly different in our study. Considering that these studies included patients who already had severe COVID-19 on admission, it is possible that high levels of D-dimer and NLR are the result of disease severity and are not risk factors for disease progression in patients without any symptoms or those with mild symptoms.

This study has some limitations. First, it is retrospective in nature and has a relatively small sample size. There were several cases with missing values, particularly in the early stage of the pandemic, and we excluded those cases and adopted complete case analysis. In multivariate analysis, we included all factors and applied backward elimination to maximize the statistical power. As the number of patients who experienced the defined outcomes was small and statistically underpowered, we cannot conclude that they are critical risk factors for disease progression. However, our findings are consistent with those of previous studies from other countries, and we believe that the factors found in this study are likely to be risk factors for disease progression in Japanese patients with COVID-19. A larger-scale study is required to confirm our results. Second, the effects of treatment given after admission were not considered. Owing to the lack of information about effective treatment in the early stages of the COVID-19 pandemic, various drugs, including inhaled ciclesonide, intravenous dexamethasone, remdesivir, and unapproved investigational drugs, have been tried. Thus, a prospective controlled trial is needed to assess the effectiveness of a particular treatment.

## Conclusions

To the best of our knowledge, this is the first study in Japan to identify the risk factors for disease progression in patients with COVID-19 who exhibited no or mild symptoms on admission. Thus, clinicians should closely observe patients with COVID-19, especially those with risk factors such as old age, overweight, DM, and high serum ferritin levels, even if the patients are asymptomatic or exhibit mild symptoms.

## Data Availability

The datasets used and/or analyzed during the current study are available from the corresponding author on reasonable request.

## Abbreviations

COVID-19: Coronavirus disease 2019
BMI: Body mass index
SARS-CoV-2: Severe acute respiratory syndrome coronavirus 2
DM: Diabetes mellitus
CRP: C-reactive protein
LDH: Lactate dehydrogenase
NLR: Neutrophil-to-lymphocyte ratio
